# Nurses' Knowledge, Practices, and Barriers in Care of Patients with Pressure Ulcers in a Ugandan Teaching Hospital

**DOI:** 10.1155/2014/973602

**Published:** 2014-02-24

**Authors:** Ivan Mwebaza, Godfrey Katende, Sara Groves, Joyce Nankumbi

**Affiliations:** ^1^Mulago National Referral Hospital, Kampala, Uganda; ^2^Department of Nursing, College of Health Sciences, Makerere University, Kampala, Uganda; ^3^Johns Hopkins University School of Nursing, 525 N. Wolfe Street, Baltimore, MD 21205, USA

## Abstract

Pressure ulcers have been identified as a major burden of hospitalization worldwide, and nurses are at the forefront of prevention. The purpose of this study was to determine the nurses' knowledge and practices regarding risk factors, prevention, and management of pressure ulcers at a teaching hospital in Uganda. The study employed a descriptive cross-sectional design. Fifty-six Ugandan registered practicing nurses were sampled. A composite self-administered questionnaire and an observation checklist were utilized. The nurses had limited knowledge about critical parameters of pressure ulcers. Prevention practices were observed to be unreliable and uncoordinated related to a significant shortage of staff and logistics for pressure ulcer prevention. Nurses had poor access to current literature on pressure ulcer prevention. Translation of nurses' knowledge into practice is possible if barriers like staff shortage, pressure relieving devices provision, and risk assessment tools are addressed at Mulago.

## 1. Introduction

Pressure ulcers remain the chief complications of prolonged hospitalization, specifically in situations of poor nutrition, increased moisture on the skin (e.g., incontinence), prolonged pressure, and compromised sensory stimuli [1, 2]. Pressure ulcers increase the cost of hospitalization, increase patient morbidity and mortality, and play a significant role in the spread of infection in the clinical area [3, 4]. Stage IV pressure ulcers have a high cost, and stopping the progression of early stage pressure ulcers can significantly reduce expenditures in resource strained facilities and decrease unnecessary pain impacting thousands of patient lives [3, 4].

The presence or absence of pressure ulcers has been generally regarded as a performance measure of quality nursing care and overall patient health [5]. On average, 60,000 people die each year worldwide due to pressure ulcer related causes [6]. The prevalence of pressure ulcers in European hospitals ranges from 1% to 11% in medical wards and 4.7% to 66% in surgical wards [7]. A study conducted in the United States demonstrated that pressure ulcers can be avoided by applying simple interventions like using risk factor assessment scales and regular turning of the patient by the nurses or by the nurse instructing a care taker [7].

In Uganda, little or no evidence is known about the capacity of nurses in regards to best practices with pressure ulcer care and prevention. Mulago Hospital remains the largest National Referral and Teaching hospital located in Kampala with a capacity of 1500 beds. The nurses form the largest number of health care employees (852) at Mulago Hospital. Nurses who work at this hospital are from various institutions of training with different educational levels (diploma and bachelor level), and all are registered with the Uganda Nurses Council. This study provides current evidence about Mulago Hospital nurses' knowledge and practices as regard to risk factors, prevention, and management of pressure ulcers. In addition barriers to care of patients with pressure ulcers are identified.

## 2. Methods

### 2.1. Design

This was a descriptive cross-sectional quantitative study that employed nonprobability sampling technique. The study was approved by the internal review boards of the School of Health Sciences, Makerere University, and Mulago Hospital (REC REF 2012-022). Each of the participating nurses was required to sign a consent form before participating in the study.

### 2.2. Setting and Sample

The investigators recruited nurses who provided bedside care from three medical wards, three surgical wards, the burns ward, and the orthopedic ward of Mulago Hospital. This is the National Referral and Teaching Hospital with more than 400 bed capacity. These wards were identified as having the highest number of patients with pressure ulcers. A list of nurses working on the selected wards was obtained from the charge nurses and further crosschecked to exclude nurses on leave during the data collection period. All 84 nurses providing direct care on the day shift (8 am to 3 pm) in the selected wards were included in the sample.

### 2.3. Study Procedure

Nurses present on the selected wards were asked to complete a structured questionnaire after signing the consent form. No Internet access or reference materials were allowed on the ward to ensure that the nurses did not seek any external assistance to answer the questions. Of the 84 questionnaires distributed, 56 (66.6%) questionnaires were completed and included in the analysis.

A research assistant, utilizing a checklist, observed two to three nurses on each ward. The research assistant had not previously interacted with the nurses, and the nurses did not know when they would be observed. This helped minimize changes the staff might make in their usual routine care if they knew they were being observed. She noted the nurses' actions, writing down their practices of prevention and management of pressure ulcers. The checklist noted the following: number of patients with at least a first degree pressure ulcer, number of times patients at risk or who had pressure ulcers were turned, availability of pressure reduction devices, treatment of skin areas at risk of developing pressure areas, moisture prevention, pressure ulcer debridement and dressing changes, consultation with colleagues in caring for patients at risk, and presence of a formal pressure ulcer assessment tool.

### 2.4. Study Instruments

#### 2.4.1. Questionnaire

The self-administered questionnaire was written in English, and contained mainly closed ended questions. English, being the national language, is used in implementing education instructions as well as in professional communications in hospitals. The questionnaire was organized in the following four sections: sociodemographic characteristics, knowledge about pressure ulcers and risk factors, current practices to prevent and manage pressure ulcers, and barriers to providing best care.

#### 2.4.2. Observational Checklist

The observational checklist included the following aspects: number of patients with at least a first degree pressure ulcer, frequency of turning patients at risks of pressure ulcers, availability of pressure ulcer reduction devices on the ward, continuous assessment and treatment of pressure areas by the nurses, moisture prevention, debridement and daily dressing of pressure ulcers, involvement of other care providers in the care for patients with pressure ulcers, and the presence of a formal assessment tool in assessing patients for the risk of pressure ulcers.

Before actual data collection, the study instruments were pretested with ten nurses in a private hospital in Kampala and adjusted accordingly. The questionnaire and the observational checklist took an average of 30 minutes each to complete.

### 2.5. Statistical Analysis

Data were analyzed using SPSS version 16 computer program. The participants' demographic characteristics, level of knowledge, and practices regarding management and prevention of pressure ulcers were analyzed using descriptive statistics. Data were reported using means, percentages, and presented in tables.

## 3. Results

The majority (91.1%) of the participants were females. The age range was 21–60 years and less than half (44%) were between 21 and 30 years. The mean age was 25 years with a standard deviation of 2.4. Additionally participants were mostly diploma graduates (83.9%) with professional experience of 1–6+ years (Table 1).

### 3.1. Nurses' Knowledge about Pressure Ulcers

Nurses were considered to have average knowledge if at least five items for each section were identified correctly. Forty-four percent were able to identify at least five items. Less than half (39.3%) of the participants were aware that pressure ulcer management requires interdisciplinary collaboration. Less than half (42.9%) did not know that pressure ulcers develop in stages (Table 1). Half (50%) of the participants did not know that pressure ulcers could lead to permanent disabilities like bone destruction.

### 3.2. Risk Factors for Pressure Ulcers Development

Nurses were asked to identify possible risk factors for pressure ulcers (Table 2). More than half (59%) of the nurses were able to identify at least three risk factors for pressure ulcers. The majority (96.4%) identified immobility as a risk factor, and 92.9% identified pressure and friction while only 28.6% identified hypoxemia and anemia (10.7%). Less than half (42.9%) of the participants identified ischemia as a risk factor, while half (50%) identified neurologic diseases as a risk factor to pressure ulcers development.

### 3.3. Strategies for Prevention and Management of Pressure Ulcers

Eight items were used to test the nurses' knowledge and practices on the strategies of pressure ulcer prevention and management. All participants identified at least one strategy used to prevent and manage pressure ulcers (Table 2). Almost all nurses (98.2%) identified regular turning of patients as a preventive measure. An equal number of the participants identified proper hydration (42.9%) and a balanced diet (42.9%) of the patient as good practices for prevention and management of pressure ulcers.

Most of nurse participants (67.9%) saw at least one new patient with a pressure ulcer on their ward every month, and 32.1% confessed that they had not been observant of pressure ulcers while admitting patients on their ward. Only 28.6% of the nurses had turned patients they found at risk of pressure ulcers on a two-hourly basis. None of the nurses reported using any risk assessment tool for pressure ulcers assessment. When asked what they did on a daily basis when caring for patients with pressure ulcers, nurses responded that they were conducting patient education (96.4%), encouraging a balanced diet (58.9%), and using pressure reduction devices (33.9%) (Table 3).

Analysis from checklist utilization demonstrated the nurse's mode of care for patients who had or were at risk of developing pressure ulcers. The investigators recorded the parameter as being done if they observed at least two nurses completing the task on the study unit (Table 3). The nurses did not have any formal assessment tool on any ward. Pressure relieving devices were only in use on neurology. Some devices were also found on the medical wards but were not in use. The investigators found no nursing documentation of pressure ulcers management in patient records. However, nurses on medical and surgical wards were observed involving physicians and physiotherapists in the management of patients at risk of developing pressure ulcers.

### 3.4. Barriers to Providing Evidence Based Pressure Ulcer Preventive Practices

Potential and actual barriers to carrying out pressure ulcer prevention and management (Table 4). Inadequacies of supplies for pressure ulcer management and prevention and shortages of human resource for health, particularly nurses, were the most cited barriers to carrying out appropriate pressure ulcer management. Heavy workload related to shortage of staff (94.6%) and shortage of pressure relieving devices were also mentioned. Patients were also considered a barrier when they were identified as uncooperative (62.5%). Other barriers were poor access to pressure ulcer literature (37.5%) and inadequate coverage about pressure ulcers during training (23.2%) (Table 5).

## 4. Discussion

The practicing nurses who participated in the study generally had inadequate knowledge about how pressure ulcers developed. They also did not have current knowledge on how to stage the pressure ulcers, nor did they know the prognosis of unpreventable and unmanaged pressure ulcers that often lead to permanent disability and bone destruction. They had inadequate understanding of the importance of interdisciplinary management. Similar findings were also reported in spinal cord injury units in the United States [8]. According to Louis (2007) knowing that pressure ulcers develop in stages was important in prediction, prevention, and their management to determine patient care outcomes.

Participants had some level of knowledge about risk factors although fewer knew about systemic risk factors such as hypoxemia, anemia, ischemia, and neurologic diseases. It is contended that pressure ulcer management is one of the nursing activities that require an interdisciplinary approach [9]. The interdisciplinary team involves in addition to a nurse, a patient educator, wound care physician, physiotherapist, and a dietician [8]. Pressure ulcer management and prevention also involves diet, physical exercise, wound care, and patient education. The nurse who is the main care provider should be knowledgeable and in position to mobilize the care team. Participants had inadequate knowledge about the need for a balanced diet among patients at risk. This was also true among nurses in Belgium [10]. Nutrition is even more important with the geriatric patients [3].

Teaching a patient about pressure ulcer prevention is paramount and is the best practice in patient management. Firstly it empowers the patient to follow instructions given by the care nurse and, secondly, it increases patient readiness to collaboration with the health care team. Additionally, keeping the skin and beddings of the patient dry is another important prevention intervention that nurses implemented and this is consistent with what Italian nurses consider as nursing activities [11]. This practice might not be directly targeting pressure ulcers alone, but also well documented that it is a good prevention measure [12, 13].

Only 12% of nurses in selected United States hospital turned their patients every two hours [14]. This was also reflected in the practice of the nurses in this study. In another study [10] substantive tissue damage on bony prominences took place during the first 3 to 4 hours of pressure, confirming the importance of turning patients regularly. In addition, in a study [15] performed on postsurgical patients, it was found that patients who were not turned developed first stage ulcers in just two hours. In resource limited settings like Uganda regular turning of the patient may be the only available means to reduce the incidence of pressure ulcers, a practice undertaken by the patients' care taker under the supervision of the nurse.

One of the extrinsic risk factors for pressure ulcer development was pressure on bony prominences [16–20]. Prevention should be one of the health care teams' priorities and in this study only a few nurses used pressure relieving devices on their wards. This is different from findings in a study in Belgium [17] where 69.2% of all patients at risk had pressure relieving devices. This study revealed that nurses were also not using a pressure ulcer risk assessment tool and were not even aware of the existence of such tools. This is significantly different from a study done in Ireland [16] where 95% of nurses were using the assessment tool, and 75% of the staff nurses could identify which tool was being used. In another study among Korean hospitals [21] 67% of staff nurses used pressure ulcer risk assessment tools. The importance of using a risk assessment tool in predicting pressure ulcers cannot be underemphasized [22]. In addition a good number of staff nurses had not consulted with any other health professional while taking care of patient at risk or with pressure ulcers. This may affect proper pressure ulcer care management of patients in the interdisciplinary approach; better outcome is expected [22, 23].

## 5. Implications for Nursing Practice

With noticeable increase in chronic diseases, trauma, and increasing number of aging population, nurses are required to be in position of providing pressure ulcer care and prevention. Their capacity to manage pressure ulcers needs to be enhanced through continuing education with support of the nursing administrators. Additionally, protocols to guide pressure ulcer care need to be developed and disseminated for use during care.

## 6. Recommendations for Future Research

There is need to carry out research based on the available risk assessment tools so as to identify individuals prone to pressure ulcers so that prevention and management is predictable. It would be imperative for the nurses to know, adopt, and implement evidence based risk assessment tools such as the Braden scale and also development or adoption of existing pressure ulcer protocols such that the nurses would not need to guess at the best strategies for pressure ulcer prevention and rapid healing.

## 7. Conclusions

The prevention and management of pressure ulcers is of great importance. However, nurses at Mulago Hospital have given it low priority stemming from inadequate knowledge and heavy workload such as one nurse having many patients to attend to. The nurse training schools and universities need to examine their curricula to address issues related to pressure ulcers prevention and treatment. Hospitals also need to devote more resources to prevent and manage pressure ulcers. Professionals should also meet their responsibility to provide continuous nursing education (CNE) and continuous medical education (CME) to staffs about pressure ulcers. Included and reflected in this education should be the importance of interdisciplinary collaboration.

## Figures and Tables

**Table 1:** Demographic characteristics of study participants.

Demographic characteristics	Category	%
Sex	Female	91.1
	Male	8.1

Age in years	21–30	44
	31–40	35.7
	41–50	10.7
	51–60	8.9

Level of qualification	Diploma	83.9
	Degree	16.1

Years of practice	1–3	33.9
	4–6	17.8
	>6	39.2

Ward of practice	Neuro	3.6
	Medical	48.2
	Orthopaedic	5.4
	Spinal	14.3
	Surgical	28.6

**Table 2:** Nurses knowledge about pressure ulcers.

Nurses knowledge about pressure ulcers	%
Facts about pressure ulcers	
Areas of skin compromised as a result of unrelieved pressure	83.9
Occur in immobile patients	92.9
Develop in stages	42.9
Commonly occur around bony prominences	91.1
Management requires interdisciplinary collaboration	39.3
Can lead to permanent disabilities like bone destruction	50
Sepsis is one of the complications	89.3
Contributes to overall hospital costs incurred by patient	69.6
Risk factors for developing pressure ulcers	
Immobility	96.4
Pressure/compression	92.9
Friction/shear	64.3
Hypoxemia	28.6
Malnutrition	66.1
Anemia	10.7
Ischemia	42.9
Neurologic disease	50
Strategies used in prevention	
Regular turning of patients	98.2
Keeping patients skins dry and moist	91.1
Encouraging patients to have a balanced diet	42.9
Ensuring patient is well hydrated	42.9
Removing any tightly fitting clothes from the patient	55.4
Providing cushions on areas at risk of pressure ulcers	89.3
Catheterization in case of incontinence	83.9

% indicates proportion of nurses who got the knowledge measuring item right.

**Table 3:** Nurses' daily activities when caring for a patient at risk of developing pressure ulcers.

Daily activities (multiple responses)	(*f*) %
Patient education about prevention	(53) 96.4
Pain management	(23) 41.1
Use of pressure reduction devices	(18) 33.9
Ongoing assessment of the patients	(18) 32.9
Maintaining the beddings dry	(23) 50
Keeping the patients' skin free from irritants/moisture	(44) 80.3
Encouraging balanced diet	(32) 58.9
I do not do anything about it	(17) 32

**Table 4:** Observations made during the daily nursing activities (recorded for one week on each ward studied).

Ward	Orthopedic	Medical	Surgical	Spinal	Neuro
Average number of patients on each unit	18	56	43	17	21
Number of patients with at least a first stage pressure ulcer	2 (11%)	2 (3.5%)	3 (7%)	6 (35%)	5 (24%)
Parameter observed					
Turning of patients at risk every two hours			*✓*	*✓*	*✓*
Educating a patient who is at risk or a caregiver about the prevention of pressure ulcers	*✓*	*✓*	*✓*	*✓*	
Conducting continuous assessment of areas at risk of developing pressure ulcers	*✓*			*✓*	
Controlling moisture on the skin of patients who are at risk	*✓*	*✓*	*✓*	*✓*	*✓*
Use of pressure reduction devices for patients					*✓*
Availability of pressure reduction devices		*✓*			*✓*
Involving other health workers in prevention and management of pressure ulcers		*✓*	*✓*		*✓*
Documenting and reporting about patients with or at risk of developing pressure ulcers					
Use of formal assessment tool in assessing patients for pressure ulcers					
Cleaning, debriding, and dressing of pressure ulcers on daily basis	*✓*				*✓*

**Table 5:** Barriers to carrying out pressure ulcer management and prevention (multiple responses).

Barriers	*f* (%)
Poor access to literature	21 (37.5)
Heavy workload/staff shortage	53 (94.6)
Lack of universal guidelines for prevention	28 (50)
Lack of in-service training about pressure ulcers	13 (23.2)
Uncooperative patients	35 (62.5)
Presence of other priorities other than pressure ulcers	21 (37.5)
Shortage of pressure relieving devices	45 (80.4)
Inadequate knowledge about pressure ulcers	20 (35.7)
Lack of multidisciplinary initiative	26 (46.4)
I do not have any challenge	03 (5.4)
